# Endogenous Antigen Presentation of MHC Class II Epitopes through Non-Autophagic Pathways

**DOI:** 10.3389/fimmu.2015.00464

**Published:** 2015-09-09

**Authors:** Carol S. K. Leung

**Affiliations:** ^1^Department of Haematology, University College London Cancer Institute, University College London, London, UK

**Keywords:** antigen presentation, MHC class II molecules, endogenous presentation, intracellular antigens, non-classical pathways

## Abstract

Antigenic peptides presented by major histocompatibility complex (MHC) class II molecules are generally derived from exogenous proteins acquired by antigen presenting cells. However, in some circumstances, MHC class II molecules can present intracellular proteins expressed within the antigen-presenting cells. There are several described pathways by which endogenous antigens are degraded and gain access to MHC class II molecules. These include autophagy and other non-autophagic pathways; the latter category includes the MHC class I-like pathways, heat shock protein 90-mediated pathways, and internalization from the plasma membrane. This review will summarize and discuss the non-autophagic pathways.

## Introduction

Antigenic peptides presented by major histocompatibility complex (MHC) class II molecules were thought to be mainly generated from extracellular sources, and MHC class I peptides were thought to be derived from cytosolic and nuclear proteins. In the classical MHC class II presentation pathway, exogenous antigens are acquired by antigen-presenting cells and delivered into the endo-lysosomal system, where they are broken down to peptides and presented on MHC class II molecules. While this holds true, it has become apparent that non-classical pathways also contribute to MHC class II antigen presentation. Proteins within antigen-presenting cells can endogenously access the MHC class II pathway and their constituent peptide epitopes can be presented to CD4^+^ T cells. The first few studies, shed light on the existence of an endogenous MHC class II pathway, were published in the 1980s (1–3). Jacobson et al. found that target cells endogenously expressed measles virus matrix or nucleocapsid proteins were lysed by class II-restricted measles virus-specific CD4^+^ T cell lines (4, 5), indicating cytosolic proteins could be endogenously processed and presented in the MHC class II pathway. Findings from other independent studies also corroborated this by illustrating that some viral proteins were presented by MHC class II molecules via an endogenous processing pathway (6, 7). In addition, analysis of purified natural MHC class II ligands has drawn a picture on the origins of antigens presented by MHC class II molecules. A fraction of the peptides bound to MHC class II molecules have been found to be derived from intracellular proteins (8–12). In theory, these intracellular proteins could have been liberated from apoptotic cells and recaptured as exogenous materials by the antigen-presenting cells. Nevertheless, cytosolic epitopes in these studies were isolated from cell lines that were not highly phagocytic, and a high rate of cell death and phagocytosis would be necessary to account for the abundance of some cytosolic epitopes (13). Therefore, despite exogenous antigens being the main source of antigens in the MHC class II pathway, endogenous antigens are able to access the MHC class II system. In this review, I will highlight the recent findings on endogenous MHC class II presentation pathways that are not linked to autophagy.

## Hijacking the MHC Class I Machinery

Not long after the discovery of the endogenous MHC class II presentation of antigens, components of the MHC class I pathways, such as proteasome and transporter associated with antigen-processing complex (TAP), were proposed to contribute to the non-classical presentation of MHC class II epitopes. The proteasome is a multisubunit enzyme complex that degrades a variety of proteins into short polypeptides and amino acids in the cytosol. Its role in endogenous class II presentation has been well documented (14–17). TAP was first shown to be involved in a DR1-restricted presentation and processing of a short cytosolic peptide of the influenza hemagglutinin (18), suggesting that cytosolic peptides could be imported into the endoplasmic reticulum (ER) in a TAP-dependent manner for binding to MHC class II molecules. However, as invariant chain is associated with the MHC class II molecules in the ER to prevent premature loading of peptide, the obstacle has to be overcome in this proposed pathway. Lechler and Aichinger suggested that stress conditions, such as viral infection, could alter the ratio of MHC class II molecules to invariant chain, resulting in the binding of unfolded or partially unfolded proteins to MHC class II molecules. They also proposed that high expression levels of antigen could allow antigen loading in the ER (19, 20). Indeed, it has been shown recently that unfolded proteins could compete with invariant chain and bind to MHC class II molecules in the ER before transporting to the cell surface (21). However, the same study did not find evidence that the protein–MHC class II complex on the cell surface could induce CD4^+^ T cell activation. Interestingly, there are numerous examples of CD4 epitopes being presented in the absence of invariant chain expression (22–24), and that invariant chain-negative cancer cells have been used as a means to stimulate tumor-specific T cells (25). Apart from loading the epitopes in the ER, the peptides could be loaded on internalized mature MHC class II molecules through the recycling pathway. In this pathway, the antigens may require proteasome degradation and TAP for delivering the epitopes to the early endosome, where they are loaded onto recycling MHC class II molecules. In fact, it has been demonstrated that endogenous presentation could be mediated through the recycling of MHC class II molecules (26, 27). Tewari et al. studied three I-E^d^ restricted epitopes of PR8 influenza (28) and found two of these epitopes were efficiently generated by a proteasome- and TAP-dependent pathway that involved recycling of the MHC class II molecules. Others have shown that MHC class II molecules could internalize and exchange antigenic peptides in the endosomes (29, 30), and that the peptide–MHC class II complex could rapidly recycle back to the plasma membrane through a clathrin-independent pathway (31). The conformation of the antigen is crucial for its access to the intracellular processing pathway and binding to the recycling of MHC class II molecules (32), as incompletely folded antigen could lead to selective binding to recycling of MHC class II molecules and result in CD4^+^ T cell activation.

## Regulation by Heat Shock Protein 90 (HSP90)

Recent studies on the processing of tumor-associated antigens have uncovered the involvement of components other than the MHC class I machinery in endogenous MHC class II presentation pathways. NY-ESO-1 is a cancer testis antigen expressed in a wide variety of malignant cells and spontaneous immune responses against this antigen have been detected in cancer patients (33). As CD4^+^ T cells could directly recognize MHC class II positive and NY-ESO-1-expressing tumor cells (34), one would then question the underlying mechanism of how this intracellular antigen is processed to CD4^+^ T cells. Tsuji et al. attempted to solve this by studying NY-ESO-1 epitope processing in the melanoma cell line SK-MEL-37 in the presence of various pharmacological inhibitors (35). The processing of the epitope NY-ESO-1_95–106_, restricted through DR01, was inhibited by the proteasome inhibitor epoxomicin but not lactacystin, indicating the processing of this epitope was dependent on the chymotrypsin-like activity of the proteasome. Also, tripeptidyl peptidase II and TAP were involved in degrading the protein and peptide transport. The processing required endosomal protease as treatment with chloroquine and leupeptin inhibited the presentation. However, macroautophagy and chaperone-mediated autophagy were not involved. Instead, the presentation was dependent on endosomal protease and chaperoning by the cytosolic HSP90. This chaperone protein is inducible by stress and is required for the functioning of a large number of client proteins. Both HSP90 inhibitor 17-DMAG and si-RNA-mediated silencing of the protein inhibited the CD4^+^ T cell response to epitope NY-ESO-1_95–106_, indicating the participation of HSP90 in the presentation. HSP90 has also been implicated in direct and cross antigen presentation by professional antigen-presenting cells for CD8^+^ T cell recognition (36–39); and its role in chaperoning and transferring antigenic peptides to MHC class II molecules in professional antigen-presenting cells has been described (40, 41). However, unlike the other NY-ESO-1 epitope, the DP04-restricted epitope NY-ESO-1_157–170_ was processed independent of HSP90, as treatment with the HSP90 inhibitors, 17-DMAG and redicicol as well as si-RNA-mediated silencing, had no effect in the presentation (42). This DP04-restricted presentation required TAP-mediated peptide transport and endosomal recycling. More studies are required to confirm the role of HSP90 on endogenous processing of MHC class II epitopes.

## Internalization from the Plasma Membrane

Another tumor antigen, gp100, is a melanosomal antigen that has been reported to access the endogenous MHC class II pathway and present efficiently to gp-100-specific CD4^+^ T cells (43). The presentation was inhibited by removing the putative NH_2_-terminal signal sequence and the last 70 residues in the COOH terminus in gp100, supporting the important role of melanosomal and endosomal location for efficient MHC class II presentation. In the study by Lepage and Lapointe, gp100 transfectants were used to assess the presentation of the DR07-restricted epitope gp100_170–190_. The cell-surface expression of gp100 correlated with MHC class II presentation, suggesting that gp100 might transport to the cell surface before internalizing to relevant endosomal or lysosomal compartments to meet the MHC class II molecules for presentation. The correlation between gp100 cell-surface expression and the endogenous presentation of protein was also confirmed in another study. Robila et al. analyzed the MHC class II endogenous processing of DR04-restricted epitope gp100_44–59_ and demonstrated that endosomal localization and processing by acidic proteases were necessary for gp100_44–59_ presentation (44). The major source of this epitope was from the internalization of the gp100 protein from the plasma membrane through the AP2 adaptor protein. In these studies, although the authors claimed gp100 was endogenously processed in the MHC class II pathway, their experiments could not exclude the possibility that the antigen might have been processed via a conventional exogenous route. As gp100 is a membrane-bound glycoprotein that can be secreted by cells (45), it is possible that MHC class II presentation in these studies did not result from processing of endogenous antigen, but was rather derived from exogenous antigen released from other cells in the culture. This could be easily clarified by a cell-mixing experiment, where gp100 expressing MHC-mismatched target cells are first mixed with MHC-matched target cells lacking the antigen, then co-cultured with gp100-specific CD4^+^ T cells. If CD4^+^ T cell response could be detected, then gp100 would be transferred and processed in the conventional manner. In fact, intercellular antigen transfer plays a role in the processing of MHC class II antigens for CD4^+^ T cell recognition (46, 47). However, as this pathway involves antigen being secreted and then recaptured as exogenous material by the antigen-presenting cells, this is generally considered to be conventional processing.

## Conclusion

This review has summarized the recent studies on endogenous antigen presentation of MHC class II epitopes through non-autophagic pathways. Although different pathways have been described in the non-classical MHC class II processing, there are still questions to be addressed. How are different epitopes selected for different endogenous presentation pathways to CD4^+^ T cells? Why are some epitopes better presented than the others? Despite having the same HLA restriction and epitope in a proximal region within an antigen, antigen-specific T cells display differential capability to recognize tumor cells. Tsuji et al. has shown that DR01-restricted CD4^+^ T cell lines specific for the NY-ESO-1_95–106_ epitope were able to respond to un-manipulated melanoma cell lines, while CD4^+^ T cells specific to NY-ESO-1_87–98_ could not (35). Another study also reported that two epitopes of the Epstein–Barr virus antigen EBNA1, namely VYG (EBNA1_509–528_) and PQC (EBNA1_529–548_), both restricted through DR11, behaved differently. Whereas VYG-specific CD4^+^ T cells could recognize EBV-positive LCLs directly, it was not the case for the PQC epitope (48). Poor presentation of the latter might be due to the fact that PQC is more prone to lysosomal protease degradation. However, how VYG is endogenously presented in the MHC class II pathway is still not known. In addition, most of these experiments have been *in vitro* studies; the pathways should be validated *in vivo*. Gaining a better understanding of these pathways will give insight in the development of vaccines and immunotherapies.

## Conflict of Interest Statement

The author declares that the research was conducted in the absence of any commercial or financial relationships that could be construed as a potential conflict of interest.
